# Theoretical Insights into a Near-Infrared Fluorescent Probe NI-VIS Based on the Organic Molecule for Monitoring Intracellular Viscosity

**DOI:** 10.3390/molecules28166105

**Published:** 2023-08-17

**Authors:** Yongjin Peng, He Huang, Yuling Liu, Xiaoyan Zhao

**Affiliations:** College of Bio-Informational Engineering, Jinzhou Medical University, Jinzhou 121001, China

**Keywords:** fluorescent probe, intracellular viscosity, density functional theory, electronic structure

## Abstract

So many biological functional disorders and diseases, such as atherosclerosis, hypertension, diabetes, Alzheimer’s disease, as well as cell malignancy are closely related with the intracellular viscosity. A safe and effective intracellular viscosity detecting method is desired by the biomedical community. Recently, a novel near-infrared fluorescent probe NI-VIS with a twisting intramolecular charge transfer mechanism was developed. The capability of this probe to visualize the viscosity variation in cirrhotic liver tissues and map the micro viscosity in vivo were testified using an experiment. In this work, the twisting intramolecular charge transfer mechanism and fluorescent properties of the probe NI-VIS were studied in detail under quantum mechanical method. The low energy barrier among the different conformations of the probe indicated the occurrence of twisting intramolecular charge transfer due to the rotation of the aryl group in the probe molecule while within the low viscosity environment. The electronic structure analysis on different probe conformations revealed the electron transfer process of the probe under optical excitation. All these theoretical results could provide insights into understand in greater depth the principles and build highly effective fluorescent probe to monitor the viscosity in biological samples.

## 1. Introduction

Viscosity plays an important role in all biological systems, from the microscopic level (such as cells) to the macroscopic level [1,2,3]. For viscous liquids under macroscopic conditions, it is easy to determine their viscosity with a viscometer. However, for liquids on a microscopic scale, such as in a single cell, the viscosity cannot be measured directly with a viscometer. More importantly, the micro viscosity is heterogeneous, with different components at different locations within the cell, such as cytoplasm, sub organelles, or membrane systems, having different viscosities [4,5,6,7,8,9]. Cell micro viscosity plays an important role in various biological processes, such as mass and energy transfer, signal transduction, interaction between biological macromolecules (proteins), active enzymes and cell metabolic rate, etc. [10,11,12,13,14,15,16]. Therefore, the design and synthesis of effective fluorescent probes for the determination of cell viscosity is of great significance for the study of cell physiological and pathological processes. So far, many fluorescence probes for the detection of viscosity have been reported in the literature [11,12,14,17,18,19,20,21,22,23,24,25,26,27,28].

A ratio-type fluorescent probe of viscosity was designed by Haidekker et al. [29]. They constructed a fluorescent resonance energy transfer (FRET) probe using two fluorescence groups, 2-cyano-3-(4-dimethylaminophenyl) acrylic acid methyl ester (CMAM) and 7-methoxycoumarin-3-carboxylic acid (MCCA). The fluorescence of MCCA part did not respond to viscosity changes, which could be used as the internal standard, and it was also the donor part of the FRET system. The fluorescence of the CMAM part increased with the increase in viscosity, and it was also the receptor part of the FRET system. The fluorescence of this probe had a good response to the viscosity change in the range of 1–400 mPa.s. The fluorescence emission ratio of CMAM and MCCA parts could eliminate the influence of the refractive index and dye concentration on the determination of viscosity. A BODIPY dye with a ferrocene structure as a viscosity probe was reported by Li et al. [30]. The ferrocene group was connected with the benzene ring of the BODIPY through a C-C triple bond, which could rotate freely between them, resulting in the formation of a TICT state in the electron excited state of the probe. This state attenuated to the ground state in a non-radiative way, thus reducing the fluorescence emission of probe. With the increase in viscosity, the free rotation of the C-C bond was limited, thus increasing the fluorescence emission of the probe. In the mixed solvent system of THF and ethylene glycol, the fluorescence emission of the probe increased with the increase in viscosity. The photo-physical properties and “8-Heteroaryl Effect” in 4,4-difluoro-8-(C4H3X)-4-bora-3a,4 a-diaza-s-indacene (X = O, S, Se)(BODIPY) systems were studied by Kim et al., who indicated the potential for this kind of structure to be explored as an experimental molecular fluorescent sensor applicate in life sciences [31]. The research on structural control of the photodynamics of boron-dipyrrin complexes by Kee et al. demonstrated the dominant role of aryl rotation in governing the excited state dynamics and fluorescent properties of aryl-substituted boron-dipyrrin dyes which facilitated the further use of this kind of dyes in life sciences and medical applications [32].

Miyashita et al. synthesized a green fluorescent protein (GFP) analogue to detect cholesterol content in the cellular phospholipid bilayer [33]. The probe consisted of a 4-(diphenyl) imidazolinone partially heterozygous with cholesterol. In the viscous solvent triglyceride, the fluorescence emission intensity of the probe increased with the increase in solvent viscosity. This property of the probe was also observed in the vesicles of the phospholipid bilayer. In the phospholipid bilayer, the fluorescence intensity of the probe increased with the increase in cholesterol content. A two-photon viscosity probe based on naphthalimide dye was reported by Meng et al. [34]. The fluorescence emission of this probe at 535 nm and its lifetime gradually increased with the increase in viscosity. Moreover, this probe could quantitatively detect changes in lysosome viscosity during autophagy in living cells by two-photon fluorescence lifetime imaging.

A NIR fluorescent probe NI-VIS which was utilized to create images of the mitochondrial viscosity in live cells was developed by Zhang et al. [35]. The probe NI-VIS used quinoline as an acceptor group and employed a TICT (twisted intramolecular charge transfer) mechanism to detect viscosity. A good mitochondrion targeting ability and near-infrared emission were featured in NI-VIS. The fluorescence of probe NI-VIS exhibited nearly a hundred-fold enhancement as the viscosity of a DPBS-glycerol system increased from 1.0 to 999.0 cP. Moreover, the probe NI-VIS was able to be used to map the micro-viscosity in vivo. All these results indicated that the probe NI-VIS can serve as a powerful tool to monitor the viscosity in biological samples and shows more potential in biomedical applications. The mechanism of fluorescence in probe NI-VIS responding to viscosity was studied through quantum chemistry calculation in this work. The stable structures of probe NI-VIS in ground and excited states were searched by optimization within the free energy surface. The discovered fluorescent mechanism of NI-VIS probe could be beneficial for providing insight in the design and synthetization of highly efficient fluorescent probes for application in the biomedical field.

## 2. Conformation Search

The processes of the conformation search for probe NI-VIS were as follows:

(1) Using Confab [36] to obtain initial conformations of probe NI-VIS;
(2) The batch structural optimization was conducted using Crest to invoke xtb program under the GFN2-xTB method [37];
(3) Invoke isostat in Molclus [38] program to screen out the several stable probe conformations with the local lowest free energy;
(4) The corresponding ground and excited state of different probe conformation’s structure optimization and vibrational frequency analysis on the stable probe conformations’ structure obtained from step (3) were conducted by using the ORCA program [39] under CAM-B3LYP/def2-TZVPD, a functional which was testified to be suitable for the excited state optimization with charge transfer character, with D3 dispersion correction and GCP correction to remove artificial overbinding effects from BSSE [40,41,42,43,44,45]. The functional and basis set combination wB2GP-PLYP/def2-TZVPD was used in a single point energy calculation to obtain the free energy with high precision [46,47,48,49,50,51,52,53,54]. Most of the figures in this work were rendered by means of VMD 1.9.3 software [55] and the analyses were finished by using the Multiwfn 3.8(dev) code [56].

## 3. Results and Discussion

**Figure 1:**
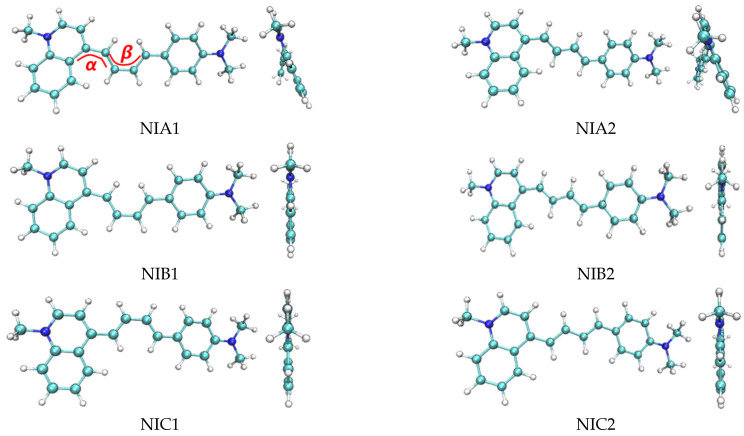
Six most stable probe conformations of probe NI-VIS.

**Figure 2:**
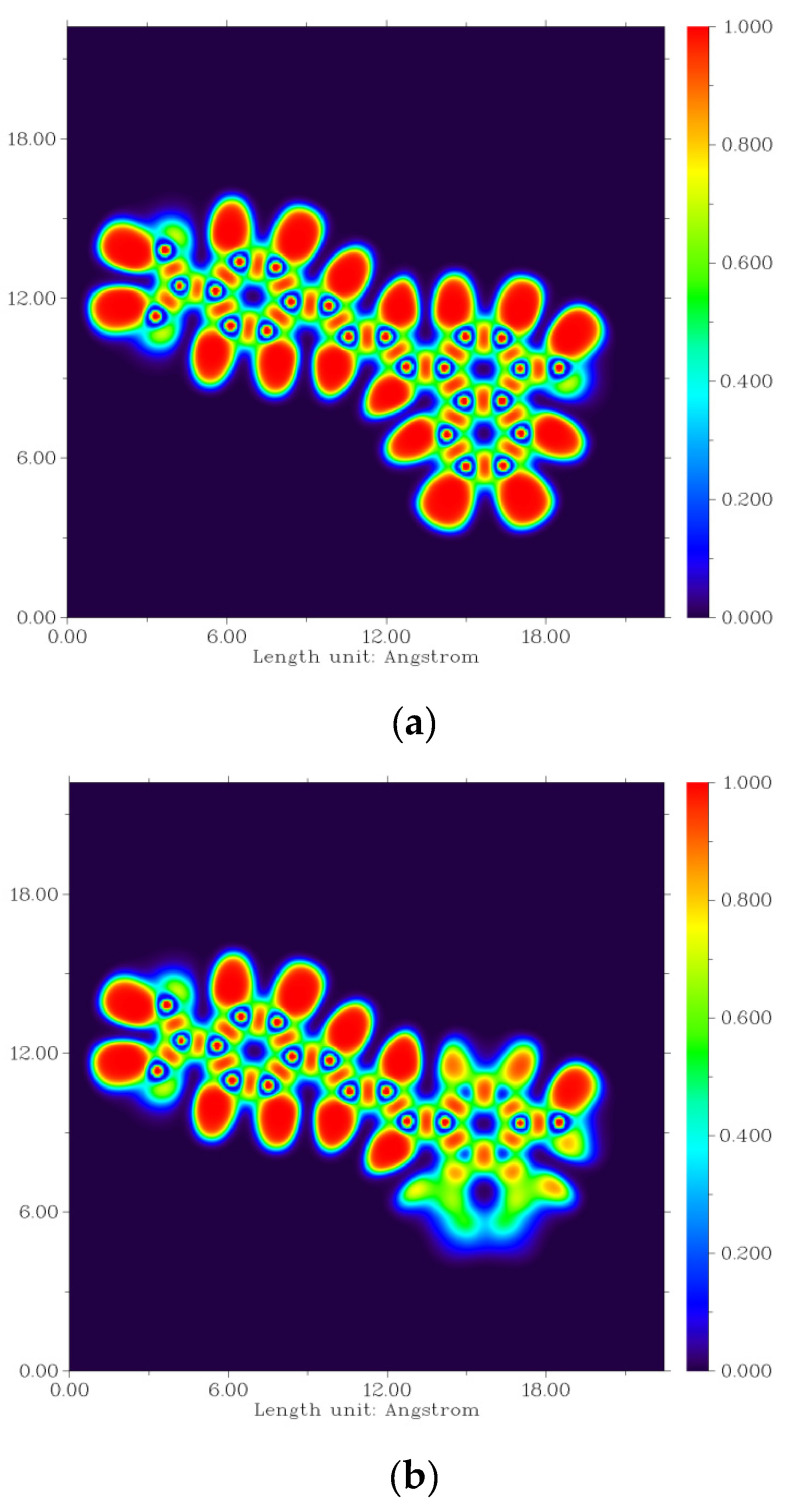
Two-dimensional plots of electron density of (**a**) NIA2 and (**b**) NIC2.

The six most stable conformations of probe NI-VIS (named by NIA1, NIA2, NIB1, NIB2, NIC1 and NIC2, respectively) are shown in Figure 1 and summarized in Table 1. It can be seen that the variation of the dihedral angle α and β led to several different stable structures with local minimum free energy. While α and β were all 180°, the corresponding structure (NIC2) had the lowest free energy which meant that there was the least repulsion effect in NIC2. When α or β were 0°, the repulsion between the adjacent H atoms resulted in the increase in the free energy of the probe conformation. To reduce the repulsion effect, the changing of α from 0° to 24° would lead to the decrease in the free energy of the probe conformation (NIA1 compared to NIB1, NIA2 compared to NIB2). The 2-dimentional plots of electron densities in NIA2 and NIC2 were depicted in Figure 2. It could be clearly shown the torsion of the dihedral angle α in the NIA2 conformation.

The most stable conformations of probe NI-VIS found here were different from the theoretical part discussed in reference [35]. In the reference the different conformations of probe NI-VIS were ascribed to the rotation of the C-C bond which connected the benzene ring and carbon chain but not the variation of the dihedral angle α and β, as shown in present study. The contradiction could be due to the incomplete basis set 6-31G*used in reference [35].

**Figure 3:**
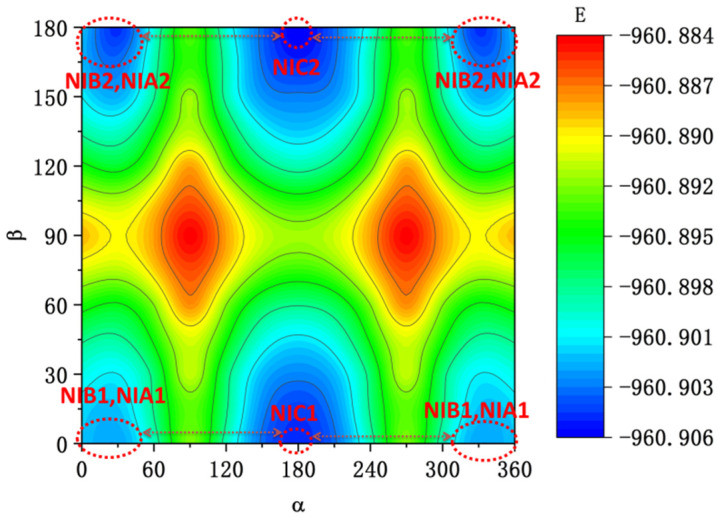
Probe NI-VIS’ structure optimization scan on dihedral angle α and β, the unit of free energy E was Hatree.

To obtain more information about the conformation difference in the probe NI-VIS, a structure optimization scan on dihedral angle α and β was conducted based on the former conformation search results. The 2D projection plot of different conformation’s free energy was shown in Figure 3. The dihedral angle scan steps were both chosen as 30° for α and β to save computational time. It could be clearly seen from Figure 3 that there were all six stable conformations with lower free energy which was consistent with the former conformation search results. The most stable conformation out of these six conformations was NIC2 with α and β both equal to 180°. The low free energy barrier, which was lower than 15 kcal/mol between different stable conformations, made the probe NI-VIS capable of taking different conformations within the low-viscosity solvent. This theoretical result was consistent with the experimental discussion in the reference [35].

**Figure 4:**
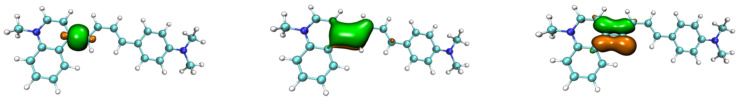
NAdO distribution of α-related C-C bond in NIC1.

**Figure 5:**
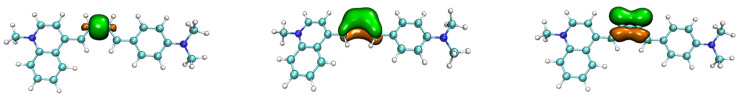
NAdO distribution of β-related C-C bond in NIC1.

**Figure 6:**
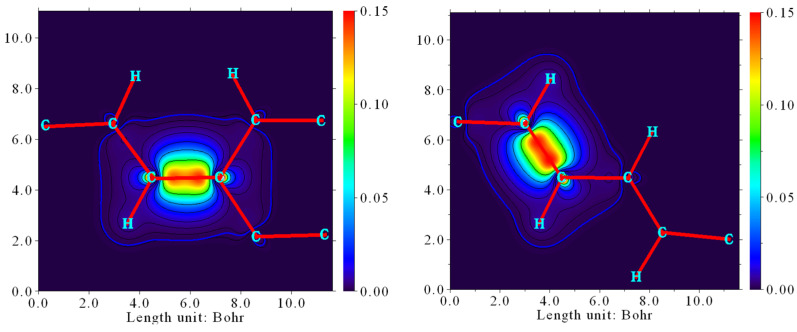
NAdO distribution 2D projection of α and β-related C-C bond in NIC1.

The natural adaptive orbital (NAdO) distribution of corresponding rotating C-C bonds within α and β were analyzed with Multiwfn 3.8 (dev). From the 3D plot and 2D projection of the two C-C bonds’ NAdO distribution, as shown in Figure 4, Figure 5 and Figure 6, it could be found that the NAdO distribution of the α and β were almost the same.

**Figure 7:**
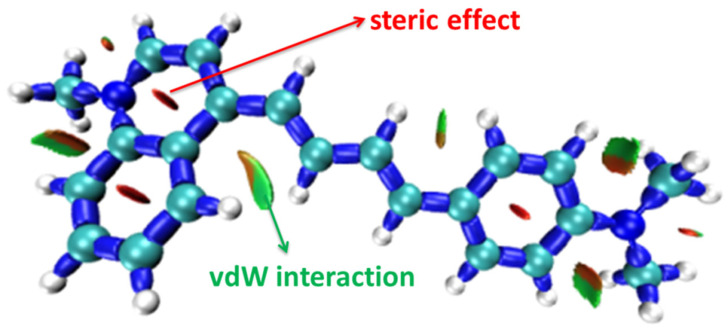
Interaction effect in NIA2.

To illustrate the interaction between atoms in the probe NI-VIS, the steric effect and vdW interaction within the conformation NIA2 analyzed via the interaction region indicator (IRI) method are depicted in Figure 7. It can be clearly seen that the variation of α from 0° to 24° enlarges the vdW interaction stabilizing the conformation NIA2 compared to the NIB2.

## 4. Densities of States (DOS)

**Figure 8:**
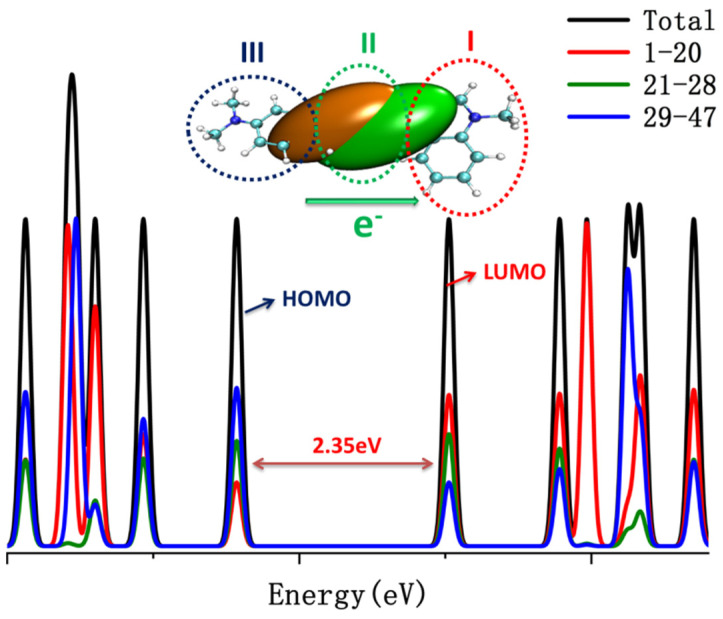
DOS of NIC2, orange and green in color, in the molecule structure inset represented the distribution center of hole and electron, respectively, three parts I II and III indicated the atom numbers 1–20, 21–28 and 29–47 respectively.

**Figure 9:**
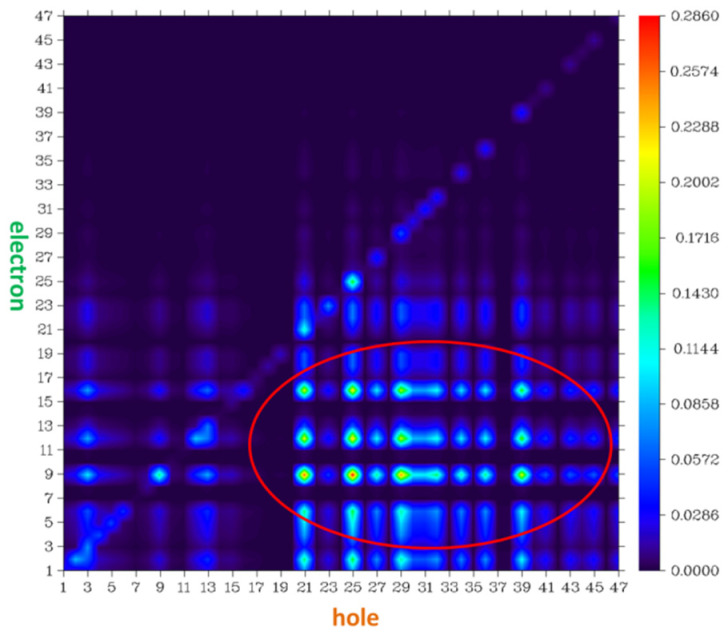
Atom–atom electron transfer heat map of probe conformation NIC2.

To understand the electron structures of probe NI-VIS, the DOS of probe NI-VIS was analyzed. It was found that all the conformations of probe NI-VIS had similar DOS structures. For clarity consideration, the DOS of NIC2 with lowest free energy was depicted in Figure 8 as an example. The whole probe molecule was divided into three parts with atom numbers 1–20 (I), 21–28 (II) and 29–47 (III) (the atom numbers can be referenced to Figures S1 and S2 in the supporting information). It can be clearly seen that the HOMO and LUMO contained the contribution of the three parts but at a different ratio. Part III contributed the most to HOMO, while part I contributed the most to LUMO. The electron transfer analysis in the optical excitation process within the probe NI-VIS also indicated the electron transfer from part III and part II to part I (quinoline), which was consistent with the DOS analysis results. From the atom–atom electron transfer analysis result (seen Figure 9), it can be seen that the electrons are mainly transfered from atom number 21, 25, 29, 39 within part III and part II to atom number 9, 12, 16 within part I.

## 5. Fluorescent Properties

**Figure 10:**
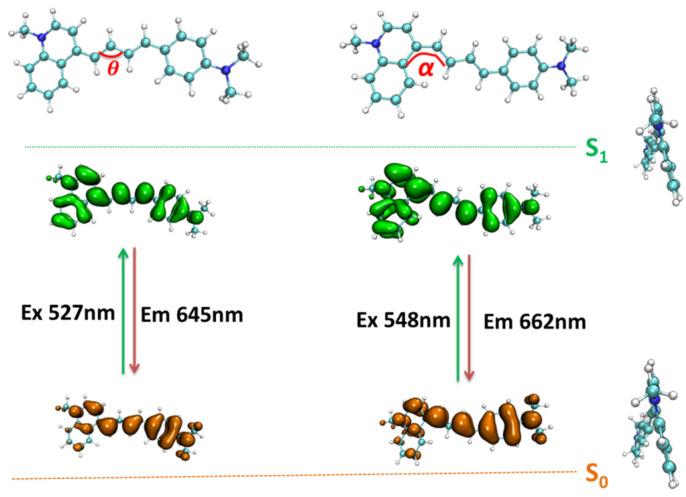
Theoretical calculations on the electron excitation and emission process of the probe molecule NIC2 and NIA1 (orange part represents a hole, green part represents an electron).

To understand the fluorescent properties of probe NI-VIS, the optical excitation and emission process of the probe were analyzed using TD-DFT methods. The corresponding structure of ground state S_0_ and several lower excited states of different probe conformations were optimized, respectively. Other than the first excited state S_1_, no other low-lying dark states were found in each probe conformation’s excited states calculation. Meanwhile the S_0_/S_1_ conical intersection point search results (see Figure S4) indicated a large energy barrier between the CI point and the stable S_1_ and S_0_ structure which would lead to a radiative decay from S_1_ to S_0_ in the DE excitation process of each probe while they could not transfer between each other, due to being in a high viscosity environment. There were slight structure variations between the ground state S_0_ and the first excited state S_1_ within all six probe conformations. Within the conformations NIA1 and NIA2, the main variation of the structure between S_0_ and S_1_ was the dihedral α. It turned from about 24° in S_0_ to about 14° in S_1_. While within the other four conformations (α, β = 0° or 180°), the main variation of the structure between S_0_ and S_1_ was the angle θ, as shown in Figure 10, in which only NIC2 and NIA1 were shown as examples for clarity. The angle θ was almost turned from 130° in S_0_ to 136° in S_1._ The electron density difference between ground state S_0_ and first excited state S_1_ of the probe molecule NIC2 and NIA1 and the electron transfer from hole part to electron part while being under optical excitation are also shown in Figure 10, which indicated a local excitation process (π-π transition) occurs when the probe molecules are optically excited. The detailed analysis of optical excitation and emission process within all six stable probe conformations are summarized in Table 2 and Table 3. It could be seen that the calculated central wavelength of excitation and emission process were about 540 nm and 650 nm, which was consistent with the experimental value reported in reference [35]. While in a low-viscosity environment, the lower energy barrier between different stable probe conformations (lower than 15 kcal/mol, as shown in Figure 3) led to an easy transfer between the different stable probe conformations, which provided a efficient non-radiative way for the DE excitation of the probe molecule and caused very low intensity of the fluorescence in this situation. The increasing viscosity of the environment would effectively cut off the transfer way between different stable probe conformations which trapped the probe molecule in the few conformations with lower free energy and led to a significant enhancement of the fluorescence just as exhibited in the experimental research.

**Figure 11:**
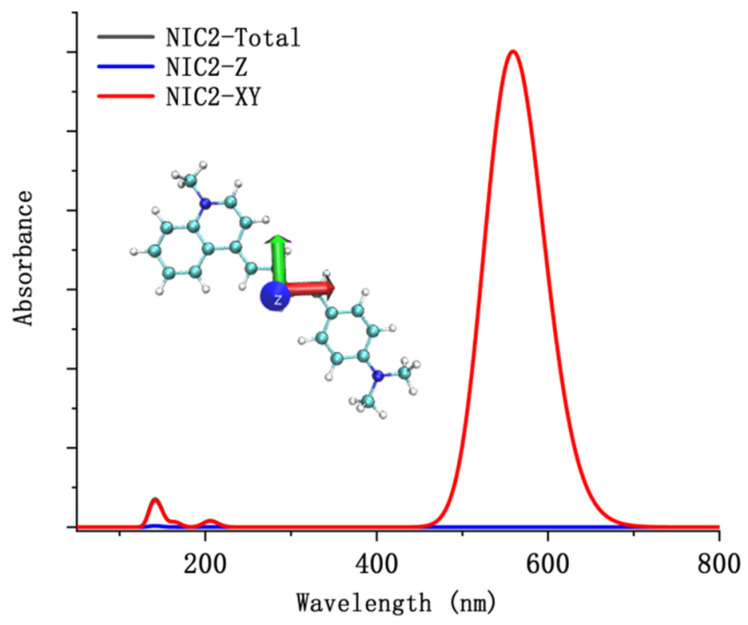
The directional UV-Vis spectrum of the probe conformation NIC2.

**Figure 12:**
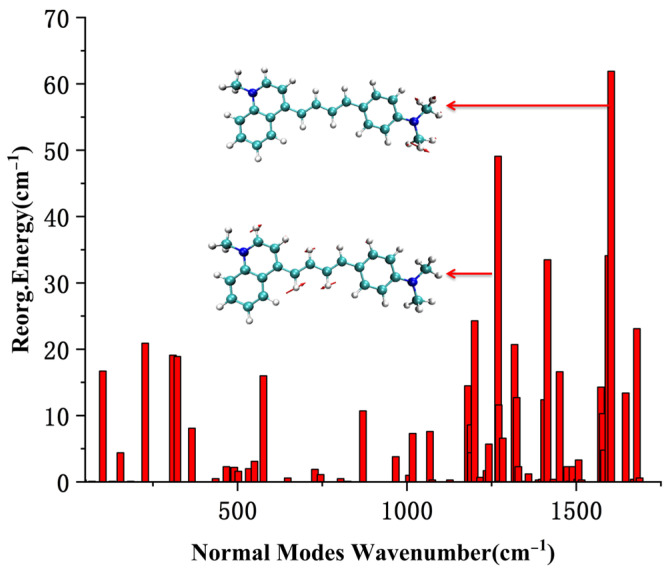
Reorganization energy of NIC2 (S_0_ to S_1_).

To further understand the character of the electron excitation process of the probe NI-VIS, the directional UV-Vis spectrum of the different probe conformations was studied using TD-DFT methods. The theoretical results indicated the main absorbed electric field by the probe NI-VIS in the excitation process lied within the probe molecular plane while the electric field which vibrated perpendicular to the molecular plane (z-axis in Figure 11) was barely absorbed by the probe molecule. Only the directional UV-Vis spectrum of probe conformation NIC2 was depicted in Figure 11 for clarity purposes. The reorganization energy between ground state S_0_ and first excited state S_1_ of different probe NI-VIS conformation were calculated. It can be seen in Figure 12 (NIC2) that the main vibration contribution to the reorganization energy was the torsion of the C-H bond. The reorganization energy between ground state S_0_ and first excited state S_1_ of NIA2 could be referenced in Figure S3 of supporting information.

## 6. Conclusions

Different stable conformations of near-infrared fluorescent probe NI-VIS were found using the quantum chemical theoretical method. The calculated low energy barrier among different probe conformations indicated the structure variation of the probe NI-VIS in the low viscosity environment, which provided the efficient non-radiative way for the DE excitation of the probe molecule and caused the very low intensity of the fluorescence in this situation. The increasing viscosity of the environment would effectively cut off the transfer pathway between different stable probe conformations, which trapped the probe molecule in the few conformations with lower free energy and led to a significant fluorescence enhancement. The analysis of the related C-C bond combined with the interaction effect indicated the C-C bond rotation was the origin of the structure variation occurring in the probe molecule. The electron transfer analysis within the optical excitation and emission process indicated that the quinolone group in the probe molecule acted as an electron acceptor. The main electric field absorbed by the probe NI-VIS in the optical excitation process lay within the probe molecular plane while the electric field, which vibrated perpendicular to the molecular plane, was barely absorbed. All the above theoretical results could provide insights to deepen our understanding and design a highly efficient fluorescent probe monitoring viscosity which could be applied in the biological field.

## Figures and Tables

**Table 1:** Parameters of six stable probe conformations.

	α	β	ΔG (kcal/mol) NIC2 Taken as Reference
NIA1	24°	0°	1.79
NIA2	24°	180°	0.88
NIB1	0°	0°	2.10
NIB2	0°	180°	1.05
NIC1	180°	0°	1.17
NIC2	180°	180°	0

**Table 2:** The main electron excitation processes in the probe molecule.

Probe	Electronic Transition ^a^	Excitation Energy	Oscillator Strength	Composition ^b^	CI ^c^
NIA1	S_0_ → S_1_	548 nm	2.0151	H → L+1 H → L H-1 → L	0.1002 0.6941 0.1124
NIA2	S_0_ → S_1_	553 nm	1.8795	H-1 → L H → L	0.1005 0.6825
NIB1	S_0_ → S_1_	537 nm	0.9696	H → L H-1 → L	0.6941 0.1127
NIB2	S_0_ → S_1_	545 nm	1.4352	H → L H-1 → L	0.6501 0.1141
NIC1	S_0_ → S_1_	518 nm	1.8625	H → L H-1 → L	0.6786 0.1064
NIC2	S_0_ → S_1_	527 nm	1.9992	H → L H-1 → L	0.6875 0.1129

^a^: Only the excited states with oscillator strength larger than 0.1 were considered. ^b^: H stands for HOMO and L stands for LUMO. ^c^: Coefficient of the wave function for each excitation was in absolute value.

**Table 3:** The main emission processes in the probe molecule.

Probe	Electronic Transition ^a^	Emission Energy	Oscillator Strength	Composition ^b^	CI ^c^
NIA1	S_1_ → S_0_	662 nm	2.1046	H → L	0.6835
NIA2	S_1_ → S_0_	658 nm	2.0135	H → L	0.6932
NIB1	S_1_ → S_0_	651 nm	2.2160	H → L	0.7067
NIB2	S_1_ → S_0_	640 nm	2.2504	H → L	0.6665
NIC1	S_1_ → S_0_	637 nm	2.2312	H → L	0.7153
NIC2	S_1_ → S_0_	645 nm	2.3014	H → L	0.7018

^a,b,c^ same indication as in Table 2.

## Data Availability

Corresponding data could be obtained on request through author’s email.

## Supplementary Materials

The following supporting information can be downloaded at: https://www.mdpi.com/article/10.3390/molecules28166105/s1, Figure S1: The stable structure of NIA2; Figure S2: The stable structure of NIC2; Figure S3: Reorganization energy of NIA2 (S_0_ to S_1_); Figure S4: S_0_/S_1_ conical intersection point search results of NIC2.
