# Supplementary material for: Theoretical Insights into a Near-Infrared Fluorescent Probe NI-VIS Based on the Organic Molecule for Monitoring Intracellular Viscosity
Source: Molecules. 2023 Aug 17;28(16):6105. doi: 10.3390/molecules28166105 (PMC10458998; doi:10.3390/molecules28166105)
Supplement: Supplementary file 1 [file molecules-28-06105-s001.zip › molecules-2420633-supplementary.pdf]

Theoretical Insights into a Near-Infrared Fluorescent Probe NI-VIS Based on the Organic Molecule for Monitoring Intracellular Viscosity

Yongjin Peng*, Yuling Liu, He Huang, Xiaoyan Zhao*

College of Bio-informational Engineering, Jinzhou Medical University, Jinzhou 121001, P. R. China

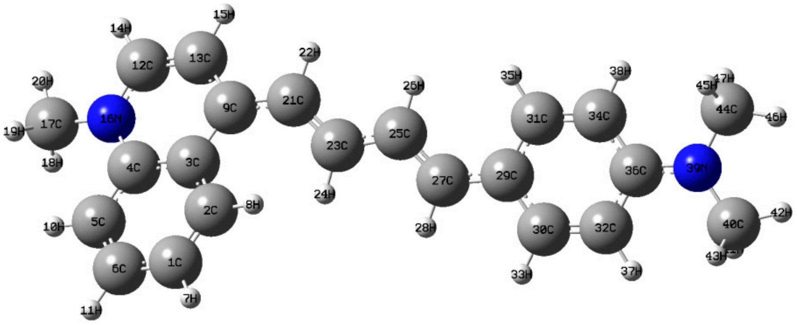

Figure S1 the stable structure of NIA2

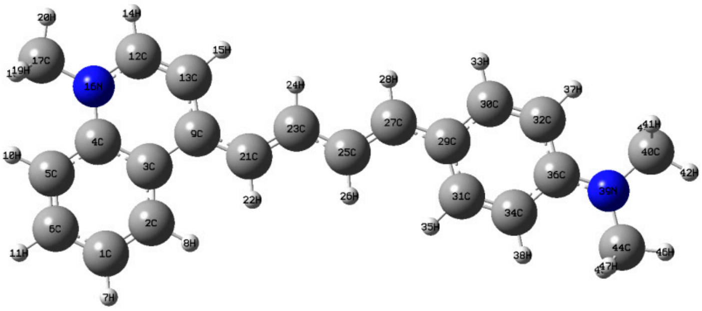

Figure S2 the stable structure of NIC2

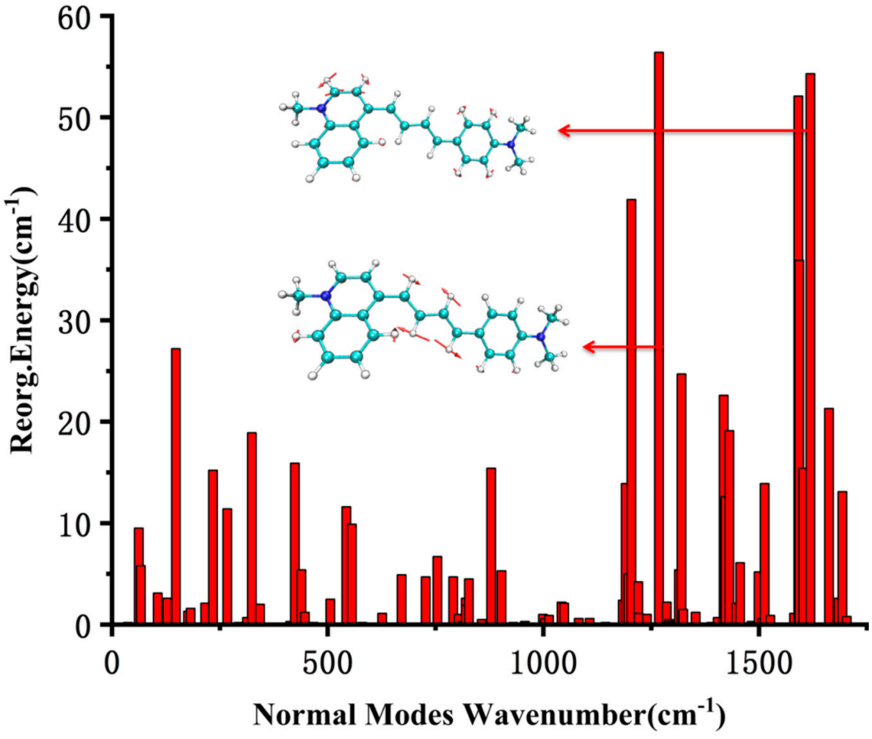

Figure S3 Reorganization energy of NIA2 (S_0 to S_1)

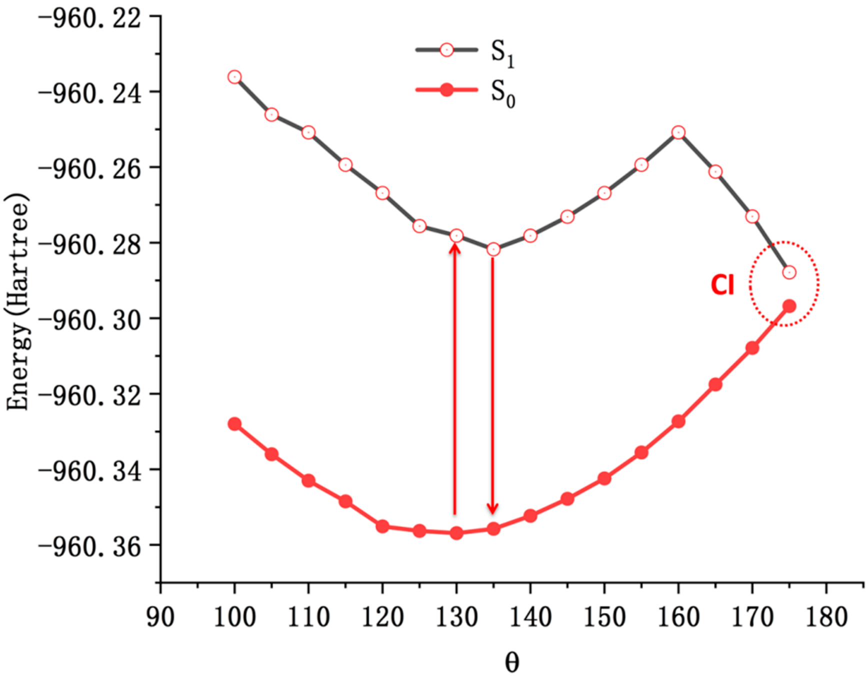

Figure S4 S_0/S_1 conical intersection point search results of NIC2

(Dr Min Feng from Nankai University was appreciated for using Gaussview to make figure S1 and S2)
